# Immunomodulatory treatment may change functional and structural brain imaging in severe mental disorders

**DOI:** 10.1016/j.bbih.2024.100864

**Published:** 2024-09-16

**Authors:** Per Thunberg, David Fresnais, Paul Hamilton, Susanne Bejerot, Mats B. Humble

**Affiliations:** a Department of Radiology and Medical Physics, Center for Experimental and Biomedical Imaging in Örebro (CEBIO), Faculty of Medicine and Health, Örebro University, 70182, Örebro, Sweden; b School of Medical Sciences, Faculty of Medicine and Health, Örebro University, Örebro, Sweden; c Department of Biomedical and Clinical Sciences, Center for Social and Affective Neuroscience (CSAN), Linköping University, Linköping, Sweden; d University Health Care Research Center, Faculty of Medicine and Health, Örebro University, Örebro, Sweden

**Keywords:** Immunopsychiatry, Schizophrenia, Obsessive-compulsive disorder, Neuroinflammation, Rituximab, Functional magnetic resonance imaging, Resting state functional connectivity, Voxel-based morphometry

## Abstract

Neuroinflammation has been implicated in the pathophysiology of schizophrenia and obsessive-compulsive disorder (OCD) and deviations in brain structure and connectivity are seen in these disorders. Here, we explore the effects of a potent immunomodulatory treatment on neuroimaging. In a pilot study of rituximab treatment in schizophrenia and OCD, a subgroup (n = 13) underwent structural and functional magnetic resonance imaging before and 5 months after treatment, to study longitudinal changes in resting-state functional connectivity (rsFC) and voxel-based morphometry (VBM).

A hypothesis-free exploratory whole-brain analysis was performed twice to assess changes in rsFC, using anterior cingulate cortex, anterior insula, posterior insula and nucleus accumbens as seed regions. There were significant interactions (diagnosis x time) in connectivity between right posterior insula and two clusters encompassing basal ganglia and anterior frontal pole, and between left anterior insula and a cluster in basal ganglia, where connectivity decreased in OCD and increased in schizophrenia. The increase of connectivity after rituximab, between left anterior insula and parts of cerebellum and lingual gyrus and between left posterior insula and parts of cerebellum, correlated with improved global psychosocial functioning according to the Personal and Social Performance Scale, especially in schizophrenia. VBM analysis identified two clusters with increased grey matter volumes (GMV) after rituximab, one in right insula overlapping one of the seed regions with significant rsFC changes. This pilot study implies that rituximab may influence both brain structure and connectivity and that GMV changes and rsFC changes are regionally associated.

## 1 Introduction

The aetiology and physiopathology of schizophrenia and obsessive-compulsive disorder (OCD) are still insufficiently understood. Schizophrenia is a heterogeneous mental disorder presenting with psychotic symptoms, and various behavioural and cognitive dysfunctions and is associated with severe disability. OCD is sometimes a similarly disabling and long-lasting disorder. The pathophysiological causes of these disorders remain unclear, which is reflected by treatment failure in approximately 30% (Howes et al., 2022).

In schizophrenia, alterations in resting state functional connectivity (rsFC) have been observed, as well as disorganized communication among large-scale brain networks (Sheffield and Barch, 2016), supporting the idea that aberrant functional integration between brain regions could be implicated (Dong et al., 2018). In OCD, altered connectivity across brain regions has also been demonstrated (Liu et al., 2022). Moreover, previous studies have shown structural differences in grey matter volume (GMV) in patients with schizophrenia (Torres et al., 2016) and OCD (Piras et al., 2015) compared to healthy controls.

The association between inflammatory mechanisms and schizophrenia is well-established (de Bartolomeis et al., 2022). Neuroinflammation has been implicated also in OCD (Endres et al., 2022). Several anti-inflammatory agents have been tested with varying results (Jeppesen et al., 2020; Grassi et al., 2020), but to our knowledge, no studies have investigated neuroimaging changes induced by potent immunomodulatory treatments in psychiatric disorders.

As regards the central nervous system, treatments with specific targets within the immune system have mainly been used in well-defined immunological disorders, e.g. autoimmune encephalitides and multiple sclerosis. Recently, however, some trials have explored their effect on psychiatric diagnoses with putative immune involvement, such as schizophrenia and OCD. Rituximab is a monoclonal CD20 antibody, one dose of which depletes B-lymphocytes and alters the immune system for at least 6 months, thereby improving inflammatory disorders, such as multiple sclerosis and rheumatoid arthritis. We intended to investigate rituximab exploratively as an adjuvant, single-dose, add-on therapy in psychiatric disorders. Still, due to safety concerns regarding this novel treatment strategy, only small open-label trials for treatment-resistant schizophrenia and OCD, respectively (Bejerot et al., 2023a), were allowed by the Swedish Medical Products Agency. Herein, we also exploratively investigated rsFC and brain structure, before and 5 months after treatment, using functional magnetic resonance imaging (fMRI) and voxel-based morphometry (VBM).

Our aim was to identify potential changes in rsFC and brain structure, possibly representing rituximab-induced changes of immune impact on brain function, thereby informing future studies.

## 2 Materials and methods

### 2.1 Subjects

Patients, 18–40 years, with treatment-resistant schizophrenia or OCD and stable treatment for at least 1 month, were recruited. Inclusion criteria were ≥2 years' disease duration, rated as “markedly ill” or worse, and severe disability according to the Global Assessment of Functioning (GAF) scale. Participants were requested to remain on their standard psychiatric treatment for schizophrenia or OCD, however current clozapine treatment was not allowed, due to its immunomodulatory effects, possibly interacting with rituximab; for details see (Bejerot et al., 2023a).

### 2.2 Study design

These two, open-label, phase 2 studies were conducted in parallel at the Örebro University Hospital between September 2019 and March 2022. One single infusion of 1000 mg rituximab was added to treatment as usual and administered according to the Rheumatology clinic's local protocol.

Comprehensive psychiatric assessments with the patients and their next of kin, also including MRI, biological sampling and cognitive-motor function test, were performed pre-treatment (Baseline) and repeated 5 months after treatment. Each participant was followed for one year.

The primary outcome measures were changes from Baseline to month 5 post-treatment in the Positive and Negative Syndrome Scale (PANSS) (Kay et al., 1987) for schizophrenia and in the Yale-Brown Obsessive Compulsive Scale (Y-BOCS) for OCD. However, to enable merging results from both diagnostic groups, we here use the Clinical Global Impression–Improvement (CGI-I), measuring the patient's change relative to baseline, ranging between 1 ‘very much improved’ and 7 ‘very much worse’) at 5 months, and changes over time in the Personal and Social Performance Scale (PSP) (Morosini et al., 2000). PSP measures global psychosocial functioning irrespective of psychiatric diagnosis, with scores ranging from 1 to 100. Low scores represent markedly impaired functioning.

### 2.3 MRI acquisition, pre-processing of data and seeds

All MRI examinations were performed using a 3.0T MR system (Discovery 750W, GE Medical Systems, WI) and a 32-channel head coil. The protocol included a structural scan (3D T1w IR-prepared fast spoiled gradient recalled echo, “BRAVO”) with the following parameters applied: TR/TE = 8.6/3.3 ms (ms), acquired voxel size of .9 x .9 × 1.2 mm; parallel imaging acceleration (ARC) factor of 2; and a fMRI acquisition (284 vol), based on a gradient echo EPI pulse sequence using the following parameters: TR/TE = 2500/17.4 ms, slice thickness 3.6 mm, no slice gap, in-plane resolution of 3.75 × 3.75 × 3.6 mm^3^ and a reduction factor of 2 (ASSET). During scanning, the patients were asked to keep their eyes open for approximately 12 min.

Structural and functional MRI images were converted to *nifti* format using dcm2niix version 1.0.20210317 (https://github.com/rordenlab/dcm2niix). Each fMRI acquisition yielded 284 vol which were pre-processed using the pipeline available in the CONN toolbox release 20.b (Whitfield-Gabrieli and Nieto-Castanon, 2012). The default settings in the pre-processing pipeline were applied including realignment, slice-timing, outlier detection, normalisation to Montreal Neurology Institute Space (MNI) and smoothing. Subsequent denoising also used the default setting and included the removal of noise components from white matter and cerebrospinal fluid areas, subject motions, scrubbing and temporal band-pass filtering. A complete description of pre-processing and denoising can be found at: https://web.conn-toolbox.org/fmri-methods/preprocessing-pipeline and https://web.conn-toolbox.org/fmri-methods/denoising-pipeline, respectively.

The analysis of rsFC as a function of rituximab treatment was performed using CONN. In the choice of seed regions, we wanted to address areas presumably involved in neuroinflammation that are also relevant to the pathophysiology of schizophrenia and OCD. We used four a priori defined seed regions: the anterior insula, due to its central role in the salience network (Palaniyappan and Liddle, 2012), the posterior insula given that this region receives interoceptive somatosensory information from the thalamus, potentially affected by an anti-inflammatory intervention (Savitz and Harrison, 2018), the nucleus accumbens, based on preclinical work showing that inflammation induces a negative affective state through modulation of ventral striatal neurons (Klawonn et al., 2021), and the anterior cingulate cortex since inflammatory challenge modulates task-related activation in this region (Harrison et al., 2009).

Masks encompassing the seed regions were obtained using the MNI labels of different brain regions found in the labels Neuromorphometrics.nii file, available in the SPM12 software package (https://www.fil.ion.ucl.ac.uk/spm/), and then imported to CONN. The MNI labels and corresponding numbers were: anterior insula left/right = 103/102, posterior insula left/right = 171/172, nucleus accumbens (left/right) = 30/23 and anterior cingulate gyrus (left/right) = 101/100.

VBM analysis was performed using CAT12 (release 12.8.1, r2043) (Gaser et al., 2022). For the VBM no a priori hypothesis was stated, instead we performed an unconditional exploration of the whole brain. Structural images acquired pre- and post-rituximab were segmented using the longitudinal pipeline and applying a smoothing filter with a Gaussian shape and FWHM of 6 mm.

### 2.4 Statistics

The rsfMRI data were analysed using a two-way within ANOVA, with the two diagnostic groups, (schizophrenia and OCD) as the first main factor, and time (pre- and post-treatment) as the second. In another analysis, merging all 13 patients into one group, change in the PSP score (post-treatment values subtracted by pre-treatment values) was used as a covariate to evaluate potential correlations (Spearman's) between changes in PSP and rsFC. For rsFC analyses we used Random Field Theory and non-parametric statistics (two-tailed unless otherwise stated), with an initial voxel threshold of p < 0.001, and then a cluster-size p-FDR threshold of p < 0.05.

The longitudinal processed VBM data was analysed using paired t-tests (two-tailed), considering all participants as one group.

### 2.5 Ethics approval and consent

The studies were approved by the Swedish Ethical Review Agency (2019-00260/2019-00256) and the Swedish Medical Products Agency (Eu-nr 2018-004618-17/2018-004619-28), and pre-registered on ClinicalTrials.gov: NCT03983031 and NCT03983018.

## 3 Results

### 3.1 Clinical outcomes

Nine patients with schizophrenia and 10 with OCD consented to the studies and were treated with rituximab. Of these, 13 (schizophrenia, n = 6; OCD, n = 7) also underwent MRI, before and 5 months after the rituximab infusion (see Flowchart, Fig. S1). The mean interval between the two MRI examinations was 152 ± 21 days. All participants were right-handed. Although psychiatric comorbidity was prevalent, none exhibited somatic neurological disorder or tardive dyskinesia. All patients with schizophrenia were treated with D_2_ blocking agents, whereas only 4/7 patients with OCD were currently using serotonin reuptake inhibitors. One patient with schizophrenia refused her long-acting antipsychotic injectable one month after inclusion, however, she was not excluded from the result calculations. For further demographic and clinical data, see Table 1 and (Bejerot et al., 2023a).

**Table 1:** Demographics and baseline clinical descriptors of included patients.

	SCZ, n = 6	OCD, n = 7
	Mean (±SD)/Count (%)	
Males/Females	4/2	3/4
Age at inclusion, years	27.7 (4.6)	26.7 (5.7)
Duration of illness, years	10.8 (6.1)	12.9 (7.2)
Body mass index, kg/m^2^	24.1 (4.1)	26.8 (6.3)
WAIS: IQ (extrapolated^a^)	97.2 (20.7)	110.0 (23.6)
Tobacco use^b^	2/6 (33%)	1/7 (14%)
Number of concurrent psychiatric diagnoses^c^	4.3 (1.6)	4.0 (2.1)
Schizo-obsessive subtype	2/6 (33%)	**-**
Comorbid autism spectrum disorder	1/6 (17%)	1/7 (14%)
CGI-Severity (0–7)	5.8 (.75)	5.7 (.76)
PANSS total (30–210)	104.5 (30.8)	
Y-BOCS total (0–40)		26.1 (6.1)
PSP total (0–100)	30.3 (11.2)	43.9 (11.0)
Number of psychotropic medications	2.5 (1.2)	3.1 (1.9)
D_2_-blocking antipsychotics	6/6 (100%)	1/7 (14%)
SSRI/SNRI/clomipramine	1/6 (17%)	4/7 (57%)
Plasma C-reactive protein, mg/L	1.2 (1.5)	1.9 (3.4)

CGI = Clinical Global Impression, OCD = obsessive-compulsive disorder, PANSS = Positive and Negative Syndrome Scale, PSP = Personal and Social Performance scale, SCZ = schizophrenia, SNRI = serotonin-norepinephrine reuptake inhibitor, SSRI = selective serotonin reuptake inhibitor, WAIS = Wechsler Adult Intelligence Scale, Y-BOCS = Yale-Brown Obsessive Compulsive Scale.

a Based on four subscales.

b Cigarettes/Snuff corresponding to more than 3 cigarettes/day.

c Psychiatric comorbidity according to the Mini-International-Neuropsychiatric Inventory (MINI).

In the schizophrenia group, the mean PANSS score diminished from baseline 105 (range 69–137) to 78 (range 35–128) 5 months after rituximab. Among OCD patients, the mean Y-BOCS decreased from 26 (range, 15–33) to 23 (range 7–32). In the merged group, the mean PSP score improved from 38 (range 16–66) at baseline to 50 (range 21–75) after 5 months. Three of 6 schizophrenia patients and 1/7 with OCD were classified as treatment responders by the clinician (CGI-I 1 or 2) 5 months after the rituximab infusion.

The two patients with comorbid autism spectrum disorder did not respond to rituximab, however, one of the two schizo-obsessive patients responded markedly (Table 1).

### 3.2 Evaluation of fMRI

When analysing pre-planned connectivities (ANOVA), no main effects were seen for either the diagnostic group factor or the time (pre-post treatment) factor. However, a significant interaction (diagnosis x time) was observed between clusters of voxels encompassing the left anterior insula and the right caudate, putamen and thalamus. A similar interaction was observed between the right posterior insula and the right basal ganglia (putamen and caudate), and right frontal pole/anterior cingulate gyrus, see Table 2. These differential changes consisted of a decrease in rsFC after treatment for the OCD patients while there was an increase in rsFC for patients with schizophrenia.

**Table 2:** Clusters with significant interactions (diagnosis x time) of rsFC changes after treatment with rituximab.

Seed (ROI)	Anatomical location of the clusters	Cluster size (#of voxels) and coordinates of peak t-statistic	Cluster-level FDR-corrected p-value
*2-way within ANOVA, pre-planned*			
Right posterior insula	- Frontal pole right - Cingulate gyrus, anterior division	369 (10, 60, 14)	.014
	- Caudate, right - Putamen, right	347 (12, 16, 8)	.014
Left anterior insula	- Caudate right - Thalamus right - Putamen right	288 (14, 16, 8)	.029
*Correlation between rsFC changes and PSP changes*			
Left anterior insula	- Cerebellum, regions 6, right, left - Lingual gyrus, right	482 (26, -66, -20)	.025
Left posterior insula	- Cerebellum, regions 3,4,5∗, right	390 (20, −34, −22)	.035
*2-way within ANOVA, post hoc*			
VBM-derived cluster #1 (Right insula and central opercular area)	- Thalamus, right and left - Cerebellum, regions 4, 5, left	426 (−14, −36, −2)	.019
	- Putamen, left - Pallidum, left - Caudate, left	283 (−16, 6, 0)	.035
	- Middle frontal gyrus, right - Inferior frontal gyrus, right	201 (52, 28, 28)	.047

Cluster location is reported as the anatomical regions mainly encompassed by the clusters. The coordinates of the peak t-statistics in the clusters are reported, together with cluster size (number of 2x2x2 mm3 voxels). FDR = false discovery rate; VBM = voxel-based morphometry. ∗As defined by the AAL atlas, available in the CONN software.

**Fig. 1:**
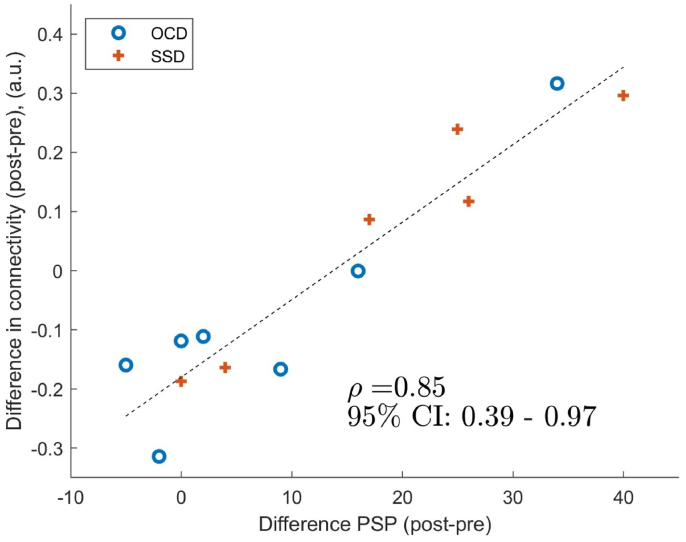
Fig. 1. Spearman's rank correlation between changes in connectivity and changes in PSP scores after rituximab treatment in patients with schizophrenia or OCD. The connectivity was measured between the seed, located in the left anterior insula, and a cluster encompassing the left and right cerebellum (parcellation 6) and the right lingual gyrus. Note that on the x-axis clinical improvement is denoted by positive values. (a.u. = arbitrary unit, OCD = obsessive-compulsive disorder, SSD = schizophrenia, PSP = Personal and Social Performance scale).

A positive correlation (Fig. 1) between changes in global functioning (as measured by PSP) and changes in rsFC after rituximab treatment was observed between the left anterior insula and a cluster encompassing the cerebellum (region 6, right and left, according to the AAL atlas) and the right lingual gyrus. Similarly, a correlation between the left posterior insula and a cluster in the right cerebellum (regions 3, 4 and 5) was observed. These correlations were observed when a one-sided positive contrast was applied.

There was a significant (p = 0.008) reduction in subject head motion (.7 ± .9 mm) during resting state measured as individual scan-to-scan difference (pre vs post) of maximum framewise displacement.

### 3.3 Evaluation of VBM

**Fig. 2:**
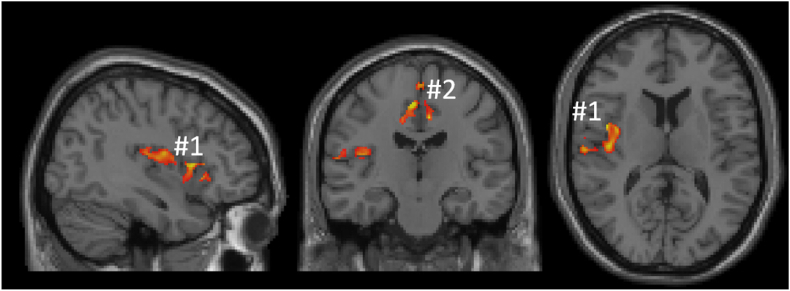
Sagittal, coronal and axial slices showing clusters with increased grey matter volume after rituximab. The cluster including the right insula (#1) is seen in all three slices while the cluster encompassing juxtapositional lobule cortex, precentral, posterior and anterior cingulate gyri (#2) is partly visualized in the coronal image.

The effect of rituximab, considering OCD and schizophrenia as one group, was an increase of GMV localized in two clusters (Fig. 2 and Table S1). One cluster encompassed right insula and central opercular area while the other included anterior and posterior cingulate gyrus, including precentral, paracingulate, anterior and posterior cingulate gyri and juxtapositional lobule cortex (supplementary motor cortex). The clusters were obtained by applying a voxelwise significance threshold level of p < 0.01 and a cluster size threshold of family-wise error(FWE)-corrected p < 0.05.

No correlations due to treatment were found between VBM and PSP and there were no differences between OCD and schizophrenia.

### 3.4 Post hoc analysis of rsFC using clusters from VBM analysis

The two clusters identified in the VBM analysis were imported to CONN and used as seed regions in a further exploratory analysis regarding changes in connectivity following rituximab treatment.

When using cluster #1 (Fig. 2) we found a connectivity interaction effect, where schizophrenia got an increased connectivity with clusters in putamen, thalamus and middle frontal gyrus, while OCD got a reduction in connectivity, Table 2.

The use of cluster #2 as a seed did not result in any significant clusters.

## 4 Discussion

In this pilot explorative study, changes in rsFC and GMV were demonstrated in treatment-resistant patients with schizophrenia or OCD after rituximab treatment, novel to the field of psychiatry. The rsFC changes observed, implied increased connectivity in schizophrenia after rituximab in contrast to OCD. Clinically, the schizophrenia patients responded markedly more favourably than the OCD patients, suggesting that rituximab may be useful as adjuvant treatment for psychotic disorders, while we cannot exclude effects also in OCD. Furthermore, the patients’ improvements in global psychosocial functioning (according to PSP) correlated positively with increased rsFC involving the left anterior and posterior insula, cerebellum and right lingual gyrus. Finally, VBM-based GMV increased in some parts of insula and cingulate gyrus. These changes took place after rituximab treatment in patients who had not responded to disorder-specific medications while continuing these medications. Therefore, the changes detected (mainly in treatment responders) are likely specific to the immunomodulatory effect of rituximab treatment. Plasma CRP was far below conventional cut-off already at baseline (Table 1) and changes after treatment did not correlate with clinical outcome. Accordingly, rituximab may have targeted an inflammatory process in the brain that does not affect circulating CRP (which is produced in the liver).

Changes in rsFC have been reported in response to treatment with serotonin reuptake inhibitors and antipsychotics in these disorders, respectively (Shin et al., 2014; Maximo et al., 2024). However, to the best of our knowledge, no previous study of psychiatric disorders has investigated changes in rsFC or VBM in response to immunotherapy. In the present study, where all patients with schizophrenia already were on antipsychotic treatment, the association between clinical outcomes and imaging changes was most noticeable in rsFC in the left insula and the VBM increase in the right insula. The insula has been implicated in the pathophysiology of schizophrenia and OCD, with studies reporting both structural (Goodkind et al., 2015; Shepherd et al., 2012) and functional changes (Tian et al., 2019; Zhu et al., 2016). In schizophrenia, insular rsFC changes have been observed in response to treatment with both antipsychotics (Duan et al., 2020) and electroconvulsive therapy (Jiang et al., 2019). Interestingly, in our study, similar rsFC changes are assumed to be the result of immunomodulatory treatment, supporting the involvement of insula in neuroimmune mechanisms of schizophrenia.

In this study, the structural changes after rituximab coincided regionally with the connectivity changes, i.e. insula and cingulate gyrus. Previous studies have shown that GMV changes are associated with connectivity alterations in schizophrenia (Corradi-Dell’Acqua et al., 2012) and OCD (Xu et al., 2023). It is not clear what the increased GMV represents but it has been assumed to be associated with cellular changes, e.g. the number of astrocytes (Asan et al., 2021). The VBM changes in this study are based on a small population with significance levels not as stringent as desired (Eklund et al., 2016), i.e. a voxelwise threshold of p < 0.001 and a cluster FWE of p < 0.05. It should be kept in mind that a significant cluster has a low spatial specificity, meaning that there are significant voxels somewhere within the cluster. Thus, in larger clusters encompassing different brain regions it is not possible to state that all regions or a specific region is affected (Nichols, 2012; Woo et al., 2014).

Our study explored an entirely novel treatment in psychiatry and suggested changes of brain structure and connectivity in psychiatric patients, responding to immunomodulatory treatment with rituximab. An ongoing study, including a larger sample size with schizophrenia, will enable further exploration of neuroimaging changes and expand on these preliminary results (Bejerot et al., 2023b).

### 4.1 Limitations

The number of patients was restricted by ethical considerations rather than statistical power calculations; thus results should be interpreted with caution. Due to the small sample size covariates were not considered. Although only 13 of the original 19 patients participated in this MRI study, biasing factors were not evident. Age, duration of illness, BMI, antipsychotic treatment and number of concurrent psychiatric diagnoses were similar to the original cohort. However, C-reactive protein in plasma differed in the schizophrenia group, i.e. 1.2 in this cohort compared to 4.1 in the original cohort. Also, a lower proportion of females participated in the MRI study compared to the original cohort.

Due to the explorative nature of the study, we did not correct for multiple testing in all calculations. Only in confirmatory studies this is required (Bender and Lange, 2001).

## Funding

This research was funded by: Nyckelfonden, grant number OLL-878311 and OLL-779081, Torsten Söderbergs stiftelse, grant number M84/19, and Hjärnfonden (The Swedish Brain Foundation), grant number FO2019-0094.

## CRediT authorship contribution statement

**Per Thunberg:** Writing – review & editing, Visualization, Validation, Software, Methodology, Investigation, Formal analysis, Data curation, Conceptualization. **David Fresnais:** Writing – review & editing, Writing – original draft. **Paul Hamilton:** Writing – review & editing, Methodology. **Susanne Bejerot:** Writing – review & editing, Resources, Project administration, Investigation, Funding acquisition, Conceptualization. **Mats B. Humble:** Writing – review & editing, Visualization, Supervision, Formal analysis, Data curation.

## Declaration of competing interest

None of the authors have any interests to declare.

## Data Availability

Data will be made available on request.
