# Supplementary material for: Immunomodulatory treatment may change functional and structural brain imaging in severe mental disorders
Source: Brain Behav Immun Health. 2024 Sep 16;41:100864. doi: 10.1016/j.bbih.2024.100864 (PMC11439893; doi:10.1016/j.bbih.2024.100864)
Supplement: Multimedia component 1 [file mmc1.docx]

Fig S1. Flowchart of patients included in the fMRI study.

Table S1. The clusters of significant differences in voxel-based morphometry (VBM) before and after treatment with rituximab are presented in the table below. The location of a cluster is reported by specifying the anatomical regions that were mainly encompassed by the cluster together with the coordinates (MNI-space) of peak t-statistics in the cluster. Cluster size is expressed as the number of 1.5x1.5x1.5 mm^3^ voxels.

The clusters were obtained after a paired t-test having a cluster-defining threshold of p<0.01 and a cluster family-wise error (FWE) corrected threshold of p<0.05.

| **Cluster** | **Anatomical location of cluster (Right or Left hemisphere)** | **Coordinates of peak t-statistic (MNI-space)** | **Size  (number of voxels)** |
| --- | --- | --- | --- |
| #1 | Central opercular cortex (R)  Frontal operculum cortex (R) Frontal orbital cortex (R)  Central opercular cortex (R) Insular cortex (R) | 46, -2, 8   44, 24, -3    38, -6, 16 | 1316 |
| #2 | Precentral gyrus (R) Juxtapositional Lobule cortex (formerly supplementary motor cortex) (R) Cingulate gyrus, posterior division (R)  Cingulate gyrus, anterior division (R) | 6, -20, 51     9,26,22 | 2442 |
